# dBMHCC: A comprehensive hepatocellular carcinoma (HCC) biomarker database provides a reliable prediction system for novel HCC phosphorylated biomarkers

**DOI:** 10.1371/journal.pone.0234084

**Published:** 2020-06-04

**Authors:** Yen-Wei Chu, Ching-Hsuan Chien, Mei-I Sung, Chi-Wei Chen, Yu-Ting Chen

**Affiliations:** 1 Institute of Genomics and Bioinformatics, National Chung Hsing University, Taichung, Taiwan; 2 Biotechnology Center, National Chung Hsing University, Taichung, Taiwan; 3 Agricultural Biotechnology Center, National Chung Hsing University, Taichung, Taiwan; 4 Institute of Molecular Biology, National Chung Hsing University, Taichung, Taiwan; 5 Ph.D. Program in Medical Biotechnology, National Chung Hsing University, Taichung, Taiwan; 6 Department of Computer Science and Engineering, National Chung-Hsing University, Taichung, Taiwan; King Faisal Specialist Hospital and Research Center, SAUDI ARABIA

## Abstract

Hepatocellular carcinoma (HCC), which is associated with an absence of obvious symptoms and poor prognosis, is the second leading cause of cancer death worldwide. Genome-wide molecular biology studies should provide biological insights into HCC development. Based on the importance of phosphorylation for signal transduction, several protein kinase inhibitors have been developed that improve the survival of cancer patients. However, a comprehensive database of HCC-related phosphorylated biomarkers (HCCPMs) and novel HCCPMs prediction platform has been lacking. We have thus constructed the dBMHCC databases to provide expression profiles, phosphorylation and drug information, and evidence type; gathered information on HCC-related pathways and their involved genes as candidate HCC biomarkers; and established a system for evaluating protein phosphorylation and HCC-related biomarkers to improve the reliability of biomarker prediction. The resulting dBMHCC contains 611 notable HCC-related genes, 234 HCC-related pathways, 17 phosphorylation-related motifs and their 255 corresponding protein kinases, 5955 HCC biomarkers, and 1077 predicted HCCPMs. Methionine adenosyltransferase 2B (MAT2B) and acireductone dioxygenase 1 (ADI1), which regulate HCC development and hepatitis C virus infection, respectively, were among the top 10 HCCPMs predicted by dBMHCC. Platelet-derived growth factor receptor alpha (PDGFRA), which had the highest evaluation score, was identified as the target of one HCC drug (Regorafenib), five cancer drugs, and four non-cancer drugs. dBMHCC is an open resource for HCC phosphorylated biomarkers, which supports researchers investigating the development of HCC and designing novel diagnosis methods and drug treatments. **Database URL:** http://predictor.nchu.edu.tw/dBMHCC.

## Introduction

Liver cancer is the sixth most common cancer in the world but is the second leading cause of cancer death worldwide [1]. Most (70% to 90%) primary liver cancers are hepatocellular carcinoma (HCC), which presents with unnoticeable symptoms, resulting in a poor prognosis and a 5-year survival rate of only 12–15% [2]. To date, surgical procedures, radioactive particle implantation, radiofrequency ablation, hepatic artery chemoembolization, and chemotherapeutics are the most popular therapy strategies for patients in early-stage HCC. Recently, a few tyrosine kinase inhibitors have been developed as chemotherapeutic agents, of which sorafenib is the only systemic therapeutic approved by the FDA and has exhibited notable survival benefits for patients with HCC [3]. Genome-wide molecular biology studies will allow us to better understand the genetic mechanisms of liver cancer and provide new options for treatment.

Reversible post‐translational modifications of proteins have a crucial role in cellular signal sensing and transduction networks, as they influence enzymatic activity and protein interactions, stability, and subcellular location. Recently, more than 200 post‐translational modifications have been described, including the addition of functional groups (e.g., phosphorylation, acetylation), chemical modification of amino acids (e.g., citrullination, carbamylation), and structural changes (e.g., disulfide bridges, proteolytic cleavage) [4]. Among them, phosphorylation is the most common and well-studied, as one-third to one-half of all proteins in eukaryotic cells are estimated to be phosphorylated [5]. Protein phosphorylation, which is regulated by protein kinases and protein phosphatases, is involved in almost all cellular processes, including cellular energy balance, antioxidant mechanisms, cellular defense, mitochondrial biogenesis, growth, differentiation, metabolism, and apoptosis. Because of its key role in maintaining the cellular condition, a bifunctional protein kinase mediated signaling pathway may influence the development of many disease conditions, especially cancers, cardiovascular disease, neurodegenerative syndromes, and rare diseases [6]. Several protein kinase inhibitors do improve the survival and quality of life of cancer patients [7]. Hypervascularization is a key component of tumor growth and metastasis. The vascular endothelial growth factor (VEGF), basic fibroblast growth factors (bFGFs), platelet-derived growth factor (PDGF), and transforming growth factor beta (TGFB) are the major angiogenic factors. These growth factors along with their receptor tyrosine kinases (RTKs) and the related RAS/RAF/MEK/ERK and PI3K signaling pathways have become major targets for drug development to treat cancer [8]. That is, building a database of disease-related proteins and their target kinases should support the development of novel biomarkers for improved diagnosis accuracy and of new drug therapies in a more rapid, comprehensive, and efficient manner.

Recently, several HCC-related biomarkers have been predicted or confirmed by experiments. To organize this heterogeneous information, multiple HCC-related databases have been lunched. OncoDB.HCC was the first complex cancer genomic database, integrating data from studies of genomic aberration, gene expression, and HCC model organisms [2]. OncoDB.HCC reported 614 HCC genes/proteins with significantly differential expression in HCC tissues [2]. The Encyclopedia of Hepatocellular Carcinoma genes Online (EHCO) systematically collected 2,906 HCC-related genes from PubMed, SAGE, microarray, and proteomics data and also found 40 of 120 evolutionarily conserved and overexpressed genes that can form a highly interactive network based on comparative genomics and interactomics analyses [9]. On a larger scale, 6927 HCC-related genes, including 143 gene signatures, and their supporting evidence—consisting of 98 microarray, proteome, and genomic studies are included in Liverome [10]. Liverome also provides functions for HCC-related gene searches and comparisons [10]. The database of prognostic biomarkers and models for HCC (dbPHCC) contains 567 biomarkers (323 proteins, 154 genes, and 90 microRNAs) and their reference information, including experimental conditions and prognostic information [11]. These five databases mainly consist of high-throughput data, clinical annotations, and prognostic information, and some of them provide functional enrichment analyses, but they lack the ability to predict biomarkers and analyze phosphorylation (Table 1).

**Table 1:** Comparison of dBMHCC and other HCC-related databases.

Characteristic	dBMHCC	dbPHCC	Liverome	EHCO	OncoDB.HCC
Year developed	2018	2016	2011	2007	2007
Organism	*Homo sapiens*	*Homo sapiens*	*Homo sapiens*	*Homo sapiens*	*Homo sapiens* and mouse
Database size	5955	567	6927	2906	613
HCC biomarkers	5068	567	143	2906	613
Experimental	611	567	143	2906	613
Predicted	4457	0	0	0	0
HCCPMs^a^	1280	0	0	0	0
Experimental	203	0	0	0	0
Predicted	1077	0	0	0	0
Covers	Protein and gene	Protein, gene, and microRNA	Protein and gene	Protein and gene	Protein and gene
Pathway information	✔	✔		✔	
Online prognosis analysis		✔			
Phosphorylation analysis	✔				
Drug information	✔	**?**			
Web service	✔	✔	✔		✔

^a^ HCCPMs: Hepatocellular carcinoma phosphorylated markers.

? dbPHCC mentioned that they provide patient sample information, clinical annotation, and prognostic information, drug information and relevant web link, but drug information was not displayed on the web site.

In this study, we constructed a comprehensive database of HCC biomarkers, dBMHCC, which includes a reliable prediction system for finding novel HCC-related phosphorylated biomarkers (HCCPMs). We also provide relevant medical information as a reference for future research and the development of treatment protocols. dBMHCC is freely available at http://predictor.nchu.edu.tw/dBMHCC.

## Materials and methods

**Fig 1:**
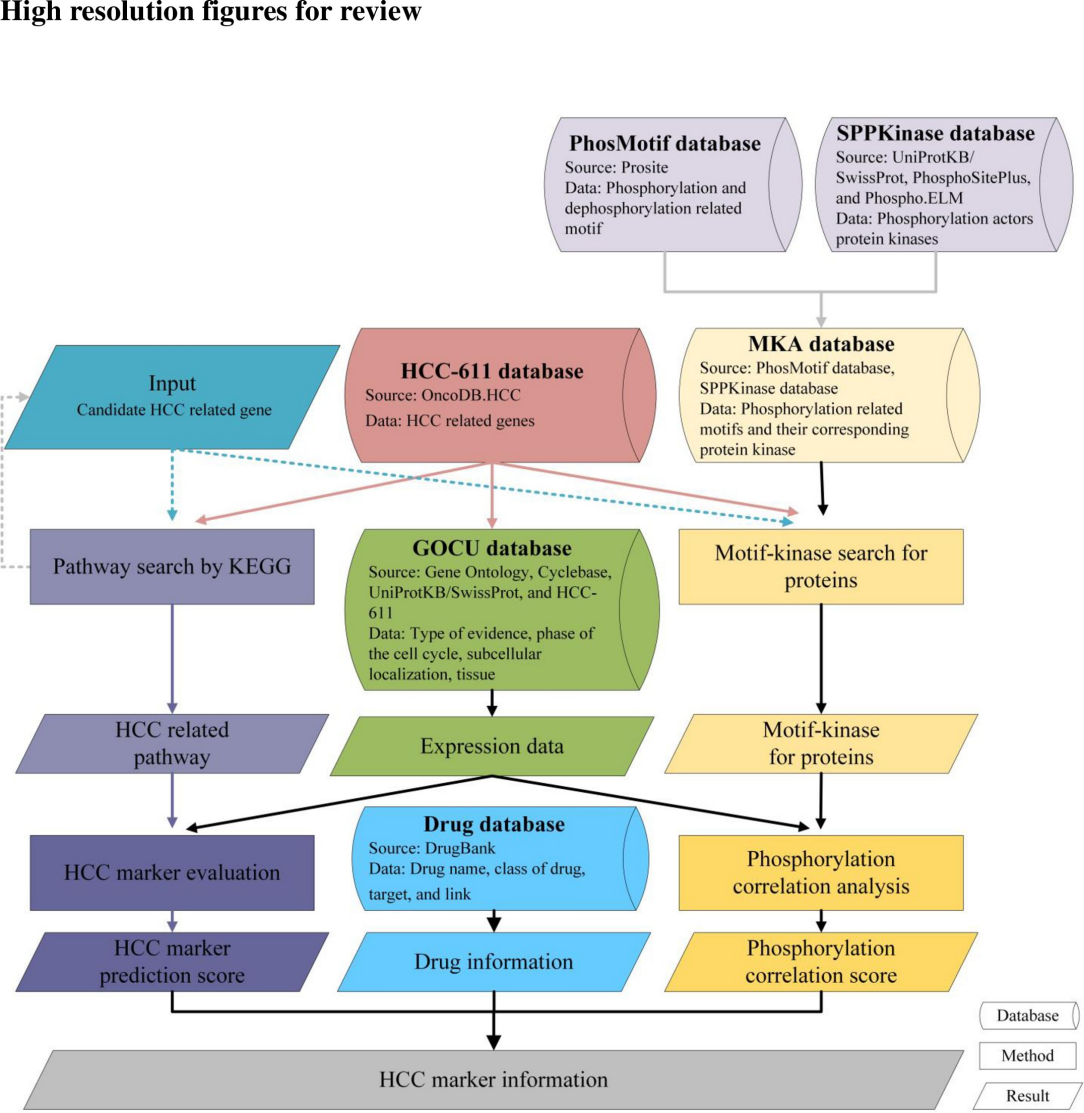
Flow chart of dBMHCC construction. The six developed databases are shown, along with the methods and resulting information used to generate the dBMHCC.

For providing a reliable prediction system to discover novel HCC-related biomarkers with phosphorylation, we constructed a comprehensive HCC biomarker database, dBMHCC (Fig 1). To achieve these approaches, we constructed six databases, HCC-611, GOCU, PhosMotif, SPPKinase, MKA, and Drug database, to gather expression profiles and phosphorylation and drug information. The MySOL has been used as database management tool for dBMHCC. For the prediction of novel HCC biomarker genes, we collected HCC-related pathways and their involved genes. We also established evaluation systems for protein phosphorylation and HCC-related biomarkers to improve the reliability of biomarker prediction. The details are described below.

### HCC-611 database construction

The information on HCC genes in HCC-611 database was obtained from “Significant gene” entries of OncoDB.HCC [2]. After updating and universalizing each gene accession number (Ensembl gene ID) and protein accession number (RefSeq accession number and UniProtKB/SwissProt accession number), the unique HCC genes were collected. For further search functions, we incorporated the corresponding National Center for Biotechnology Information (NCBI) gene ID, gene name (Ensembl gene ID), and UniProtKB/SwissProt accession number in HCC-611.

### MKA database construction

The MKA database, which integrates the information from PhosMotif and SPPKinase databases, provides phosphorylation-related motifs and their corresponding protein kinases. Based on sliding window, a comparison of each coding motif with consensus sequences is used to predict the phosphorylation function of the corresponding protein. Such motifs are good indicators for the prediction of corresponding kinases of novel HCC-related proteins. Therefore, we used “kinase OR phosph” and “phosphatase” as keywords for collecting the phosphorylation- and dephosphorylation-related motifs from Prosite [12], while constraining the search to human studies. SPPKinase database information was obtained from UniProtKB/SwissProt, PhosphoSitePlus [13, 14], and Phospho.ELM [15] phosphorylation databases. We first carried out a five-step screening process, consisting of (a) selecting human genes with experimental validation, (b) confirming the accession numbers of the related proteins, (c) finding the corresponding UniProtKB/SwissProt accession numbers for proteins collected from RefSeq, (d) judging the correctness of the protein sequences according to UniProtKB for proteins collected from RefSeq, and (e) confirming the accuracy of phosphorylation sites by Perl. The phosphorylation site should be screened for S, T, Y or H, after which the information about the corresponding kinases was presented in a uniform format.

### GOCU database construction

The GOCU database provides gene expression information, including tissue-specific information, subcellular localization of the resulting protein, expression profile during the cell cycle, and the evidence type for this information. The evidence for HCC-related genes that was obtained from OncoDB.HCC and gathered from small- or large-scale experimental analyses was classified into type E1 or E2, respectively.

To investigate novel HCC-related genes, we isolated all genes that are expressed in liver, blood, or the lymphatic system from UniProtKB/SwissProt. They were classified into three types: type T1 genes were validated by protein-level experiments, type T2 genes were validated at the mRNA level, and type T3 genes were obtained from the “Sequence origin” section of UniProtKB/SwissProt, without additional specification. Their subcellular localization information was collected from Gene Ontology [16] and UniProtKB/SwissProt. Phase information was obtained from Cyclebase [17]. After evaluating their consistency and regularity, the experimental data of first cell cycle set was retained. To allow for a consistent time period across different experiments, we divided the cell cycle into 100 time points and calculated the expression level of the gene at each time point by interpolation.

### Motif-kinase search for proteins

To predict the corresponding protein kinase, the amino acid sequences of target proteins obtained from UniProtKB were screened for phosphorylation-related motifs from the MKA database. The corresponding protein kinase of the characterized motif was defined as the corresponding protein kinase for the target protein.

### Phosphorylation correlation analysis

Considering the need for co-expression and protein interaction between a catalytic kinase and its targeted protein, we imported data about tissue and subcellular localization and the expression phase from GOCU database to carry out a phosphorylation correlation analysis. The scoring method for the tissue and subcellular localization and phase factor data was as follows:

1. Scoring for tissue (*S*_*t*_): Since this study was focused on Hepatocellular carcinoma, the genes which express in liver, blood, or the lymphatic system were collected for novel HCC-related genes prediction. In the reaction of protein phosphorylation, the catalytic kinase and its targeted protein process the phosphor group transferring in the form of protein. In order to assess the association between liver cancer-specific proteins and protein kinases, we classify proteins based on the credibility of the data source. If both genes with validation at the protein level show that they have highest chance to process the protein phosphorylation, thus the score is given as 10 (type T1). As one of them was validated at RNA level (type T2), that is, its expression in protein level was not confirmed, that reduced the credibility. Thus the score of type T2 was reduced as 6. The lowest credibility is "Sequence origin", thus if one of them was type T3 gene obtained from the “Sequence origin” section of UniProtKB/SwissProt, without additional specification (type T3), the score was as low as 2 left. In contrast, if one of the pair was not expressed in the liver, blood, or lymphatic system, there was no chance to interact and process the reaction, the score was 0.
2. Scoring for subcellular localization (*S*_*l*_): The information of subcellular localization was obtained from Gene Ontology and UniProtKB/SwissProt. Compared to UniProtKB/SwissProt, GO provided more abundance data with clear validation evidence. dBMHCC obtained 6173 and 2709 data form GO and UniprotKB/SwissProt, respectively. Due to the imbalance of the data of these two databases, a two-layer process was built for this scoring system. The first layer consisted of an analysis of the GO term from Gene Ontology and was scored based on its validation evidence. As shown in S1 Table, a gene belongs to type L1, if it has experimental evidence; type L2, if it has computational analysis evidence; type L3, if its evidence consists of a curatorial statement; or type L4, if it was automatically assigned evidence. The credibility of the validation evidence decreases in accordance with the experimental, computational analysis, automatically assigned and automatically assigned evidence. If the query protein and protein kinase are both type L1 genes, the pair is scored a 10. If one of the pair belongs to type L2 and the other is type L1 or type L2, the score will be an 8. If one of the pair is type L3 and the other is not type L4, the score will be a 4. If either of the pair is type L4, the score will be a 2. If the query protein and protein kinase belong to type L3 or L4 or are without common GO terms, with a score lower than 8, the scoring system will start the second-layer processing. Based on the subcellular localization information from UniProtKB/SwissProt, if the query protein and protein kinase have the same subcellular localization, the score of the second layer will be a 6; if they do not, the final score is simply the score from the first layer.
3. Scoring for co-expression during a phase of the cell cycle (*S*_*p*_): Co-expression is curial for protein-protein interaction, and also for the phosphor group transferring between kinase and its target protein. Thus, if the query protein and the corresponding protein kinase are both expressed at the same time point during the cell cycle, the score will be a 10; if they are not, the score will be a 0.

The kinase catalyzed phosphorylation correlation score (*S*_*k*_) between the query protein and corresponding protein kinase is calculated as in Eq (1), and the highest *S*_*k*_ is selected as the kinase correlation score.

$$S_{k}=\frac{W_{t}S_{t}+W_{l}S_{l}+W_{p}S_{p}}{W_{t}+W_{l}+W_{p}}$$ (1)

*W*_*t*_, *W*_*l*_, and *W*_*p*_ are the weight of the tissue, subcellular localization, and phase, respectively. To evaluate whether the specific protein and protein kinase can really interact in the cell, the environmental factors, including tissue and subcellular localization and the period of expression during the cell cycle. For HCC biomarker, expressing in specific tissues (liver, blood, or the lymphatic system) is the most important criteria, thus we set 5 (half of total weight) as weight of the tissue (*W*_*t*_). The subcellular localization and the period of expression during the cell cycle were needed to be considered equally. While the data size of phase was less than the data size of subcellular localization, thus we set the weight of phase (*W*_*p*_) as 2, and the weight of subcellular localization (*W*_*l*_) as 3.

### Curation of candidate HCC-related genes

We do not currently know all pathway(s) involved in the generation and development of HCC. Several studies have, however, shown that the mutation of HCC-related genes leads to a chain reaction that affects the normal physiological mechanism**s** and leads to the generation and development of HCC [18]. The Kyoto Encyclopedia of Genes and Genomes (KEGG) [19] database constructed its network based on chemical genomics information, which can provide HCC-related pathways and all the genes involved in these pathways. To look for novel candidates for HCC marker genes, we found all the related pathways in the KEGG database using a search based on HCC-611 via DBGET [20], which we referred to collectively as the HCC-related pathways. All the genes involved in these HCC-related pathways then served as candidates for HCC-related genes.

### HCC marker evaluation

HCC markers were evaluated based on the importance of their involved pathways, i.e., if more than one gene in a given pathway was experimentally confirmed to be associated with HCC. Based on the reliability of the evidence types of query gene, we developed a scoring system to assess the possibility of HCC markers as follows:

1. If the query gene belongs to HCC-611 with type E1 evidence, confirmed by wet-lab experimental data from previous reports, it has the highest credibility and its *S*_*c*_ will be set as 10. If the query gene with type E2 evidence, which is identified by microarray or proteomic reports, its *S*_*c*_ will be set as 8.
2. If the query gene was not experimentally confirmed as a HCC-associated gene, the other genes involved in the same pathway (OGSPs) are assessed. Each OGSP that is associated with type E1 evidence will be scored based on Eq (2).

$$S_{c}=W_{1}\left(\frac{N_{e}+{0.8\;N}_{m}}{N_{p}}\right)+W_{2}$$ (2)

Whereas if the OGSP was supported by only type E2 evidence, it will be scored according to Eq (3).

$$S_{c}=W_{2}\left(\frac{N_{m}}{N_{p}}\right)$$ (3)

Where *S*_*c*_ is the score for a particular HCC marker in a single pathway; *W*_*1*_ and *W*_*2*_ are the weights of a given OGSP proven by type E1 and E2 evidence, respectively; *N*_*m*_ and *N*_*e*_ are the numbers of genes proven by type E2 and E1 evidence in that pathway, respectively; and *N*_*p*_ is the number of all genes involved in the pathway. According the reliability of evidence of OGSP and limited the query gene without experimental evidence to the highest score to 8, *W*_*1*_ was given as 5, and *W*_*2*_ as 3.

Based on the influence of all the pathways that the query gene is involved in, the HCC marker prediction score is calculated as the average of weights, which will be scored according to Eq (4).

$$S_{cm}=\sum_{c=1}^{n}S_{c}W(S_{c})/\sum_{w=1}^{n}W(S_{c})$$ (4)

Where *S*_*cm*_ is the score for HCC (cancer) marker prediction. *W*(*S*_*c*_) is the weight for *S*_*c*_, and *n* is the number of pathways which the query gene involved in. If *S*_*c*_ = 10, then *W*(*S*_*c*_) = 10; if *S*_*c*_ = 8, then *W*(*S*_*c*_) = 8; if 5 ≤ *S*_*c*_ < 8, then *W*(*S*_*c*_) = 5; and if *S*_*c*_ < 5, then *W*(*S*_*c*_) = 3.

### Drug information collection

To provide the related drug information, we used DrugBank 5.0.9 [21], which includes 10,505 drugs and 16,804 targets. A gene name was used with the Targets search in DrugBank. Because the name of a gene from different resources can differ, this will influence the accuracy of the search. Thus, we downloaded the Drug Bank 5.0.9 database and used the UniProtID as the basis for our search. In addition to HCC marker prediction and phosphorylation correlation evaluation, dBMHCC also provides the drug relation by Biomarker Prediction. User can use Gene Id, Accession Number or Gene Symbol as search term by Single Search function. While user use Gene Id as criteria, that may correspond to more than one protein. dBMHCC uses all the corresponding proteins as search criteria and provide their related drug information obtained from DrugBank database, which includes identification, pharmacological information, interactions, references, clinical trial information, pharmacoeconomic data, properties, spectra, taxonomy, and other details.

## Results and discussion

### HCC-611 database

HCC-611 database collected 609 protein-encoding genes and two non-protein-encoding genes from OncoDB.HCC, and HCC-611 provided the information, including chromosome location, gene name, NCBI gene ID, and UniProtKB/SwissProt accession number for these 611 significant HCC genes (Among them, 19 entries were extracted as examples shown in S2 Table**)**.

### GOCU database

The expression pattern of a gene is an important aspect of its ability to function as a reliable HCC-related biomarker. Thus, we constructed the GOCU database for further evaluation. Considering that different experimental methods may provide different credibility, the 611 significant HCC genes were classified based on different scales of experimental analyses. In HCC-611, 421 genes had supporting evidence from small-scale analyses and were classified as Type E1, and the other 190 genes were identified based on large-scale RNA analyses and were classified as Type E2.

To narrow down the screening base of tissue information, we focused on gene expression in particular tissues, i.e., liver, blood, or lymph system, from which we isolated 18,468 proteins from UniProtKB/SwissProt (Among them, 12 entries were extracted as examples shown in S3 Table). In addition, the subcellular localization information can indicate the possibility for protein-protein interactions in the microenvironment. We obtained 17,429 proteins with 1166 GO terms from Gene Ontology (Among them, 13 entries were extracted as examples shown in S4 Table) and 6233 proteins belonging to six categories of subcellular localization, including Secreted, Cell Membrane, ER/Golgi, Mitochondrion, Cytoplasm, and Nucleus, from UniProtKB/SwissProt (Among them, 11 entries were extracted as examples shown in S5 Table). In addition, co-expression is also a key element for protein-protein interactions. We obtained 537 gene expression profiles from Cyclebase as a reference for co-expression. To allow consideration of uniform time periods from different experiments, we separated the cell cycle into 100 time points and calculated the expression level by interpolation (S1 Fig). The HCC evidence type and the co-expression profile relative to tissue and subcellular localization, and the phase of the cell cycle serve as the criteria for HCC marker and phosphorylation prediction to improve the reliability of dBMHCC.

### MKA database

The MKA database, which integrates the PhosMotif and SPPKinase databases, provides information about phosphorylation-related motifs and their corresponding protein kinases. First, we collected 252 phosphorylation-related motifs from Prosite for PhosMotif database construction. Among them, 243 are related to phosphorylation, 32 are related to dephosphorylation, and 23 have dual functions. In the dBMHCC, the PhosMotif database provides the following information: Prosite Documentation Entry, Prosite Discriminator Entry, Entry Name, Description, Pattern, Max Repeat, Site, and Skip Flag. After a five-step screening process, data about a phosphorylation site and its corresponding protein kinase**s** isolated from UniProtKB/SwissProt, Phospho.ELM, and PhosphoSitePlus were integrated into the SPPKinase database. Here information about 4161 experimentally identified phosphorylation sites from 1415 phosphorylated proteins and 348 corresponding protein kinases were collected and unified by using the UniProtKB/SwissProt accession number (Among them, 20 entries were extracted as examples shown in S6 Table). After integrating the PhosMotif and SPPKinase databases, 17 phosphorylation-related motifs were identified along with their corresponding 255 protein kinases in the MKA database (S7 Table). Among the 17 motifs, the bifunctional PS00004 and PS00018 motifs had 102 and 5 corresponding protein kinases, respectively. For the shorter patterns of the PS00004, PS00005, PS00006, and PS00007 motifs, a variety of corresponding protein kinases were identified. For example, there are 167 protein kinases that can target the PS00006 motif.

Based on the importance of protein phosphorylation in signal transduction and cellular responses, we proposed that protein phosphorylation should have a key function in HCC development. To identify the corresponding protein kinases for HCC-specific proteins, we used the genes from HCC-611 to search within MKA to find their corresponding protein kinases via motif screening. Among the HCC-611 entries, 13 phosphorylation-related motifs were found in 608 proteins, and 253 protein kinases were predicted to correspond to these 13 motifs. In the future, we may establish a BLAST tool in MKA to provide a corresponding protein kinase analysis of unknown proteins.

### Correlation analysis of phosphorylation

With MKA, we can find phosphorylation-related motifs within HCC-specific protein sequences and can search for their corresponding protein kinases, but this association alone cannot prove that the specific protein and protein kinase can really interact in the cell. Therefore, some environmental factors, including tissue and subcellular localization and the period of expression during the cell cycle, were considered for phosphorylation correlation analysis. According to the validation evidences, we built a scoring method, Eq (1). Although the query protein may be associated with several protein kinases, the highest of the kinase correlation scores is treated as *S*_*k*_. For the strongly suggestive (*S*_*k*_ ≥ 6.8), it was given as *S*_*t*_ ≥ 6, *S*_*l*_ ≥ 6, and *S*_*p*_ = 10. That is the data of tissue should be validated at the protein or RNA level, the data of subcellular localization should has experimental evidence from GO or annotated in UniProtKB/SwissProt, and the query protein and the corresponding protein kinase are both expressed at the same time point during the cell cycle. With such kinds of evidences, they are very likely to undergo a phosphorylation reaction. Considering the less data of expression profile in the phase of cell cycle, we ignore the phase criteria but keep the data with *S*_*t*_ ≥ 6, and *S*_*l*_ ≥ 6 as suggestive (6.8 > *S*_*k*_ ≥ 4.8). When the *S*_*k*_ is less than 4.8, the evidences were not sufficient to support it. We obtained 71 genes that were strongly suggestive, 1373 genes that were suggestive, and 4511 genes that were not supported. There are 24.2% of HCC-related proteins with strongly suggestive or suggestive score, based on reliable experimental evidences.

### HCC-related pathways

Based on HCC-611, we identified 391 genes involved in 234 pathways, which were then referred to as HCC-related pathways. Among these, the bladder cancer (hsa05219) pathway contains the most genes with a high level of supporting evidence: 24 genes with type E1 evidence among 38 genes in the pathway. The correlation between bladder and liver cancer development needs further investigation.

Given the complex nature of signaling networks, one gene could be involved in more than one pathway. In an extreme case, a single gene could be involved in several pathways and may function as a central hub in the development of cancer. The mitogen-activated protein kinases MAPK1 and MAPK3, which are each involved in 69 pathways (S8 Table), may be examples of this type of gene. MAPK1 and MAPK3 are the key components of MAPK signal transduction, which mediates diverse biological functions such as cell growth, adhesion, survival, and differentiation through the regulation of transcription, translation, and cytoskeletal rearrangements [22]. In particular, the MAPK1- and MAPK3-mediated Ras/MAPK pathway is significantly activated in human HCCs and is correlated with a poor prognosis [23]. Thus this kind of HCC-related gene may serve as a diagnosis indicator. As many HCC-related genes (337 of 611) are involved in <10 pathways (S9 Table), we believe that most HCC-related genes participate in specific pathways. Among these 337 HCC-related genes, 125 are involved in only one pathway. For example, the well-known oncogenic genes *NRG1*, *CD82*, *STMN1*, and *MCL1* are involved in the ErbB, p53, MAPK, and PI3K-Akt signaling pathways, respectively. In addition, *GPC3*, a member of heparan sulfate proteoglycans, has been identified as a biomarker for the diagnosis and prognosis of liver cancer [24].

### HCC marker evaluation

**Fig 2:**
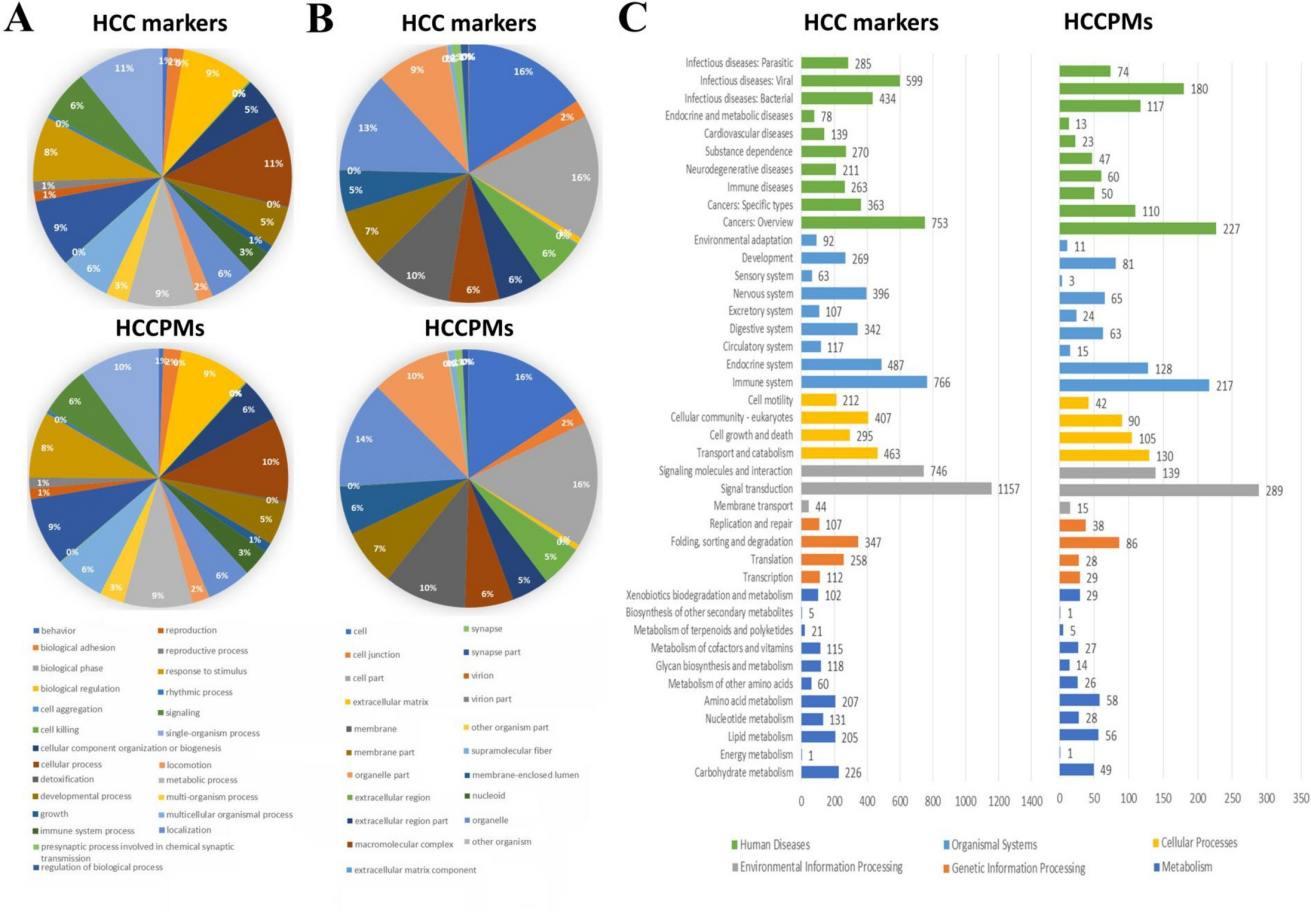
Functional analyses of HCC markers and HCCPMs. The distribution of HCC markers and HCCPMs functionally involved in Biological process (A) and Cellular component (B) was identified by GO enrichment analysis. (C) Based on KEGG analysis, the gene functions of HCC markers and HCCPMs were classified into Human Diseases, Organismal Systems, Cellular Processes, Environmental Information Processing, Genetic Information Processing, and Metabolism.

All the genes involved in the HCC-related pathways identified above (5955 genes) were further evaluated as HCC markers based on their level of experimental confirmation. The HCC markers with type E1 or type E2 evidence are known biomarkers. To investigate the novel HCC marker, we evaluated the possibility according to the importance of their involved pathways. The more genes in a pathway are verified with more credible evidence to be related to liver cancer, the more important this pathway is. Thus, for suggestive, if the query gene was not experimentally confirmed as a HCC-associated gene, all the other genes involved in the same pathway should be associated with large scale experimental evidences E2 at least (*S*_*cm*_ ≥ 3, calculated by Eq (4)). The query gene with *S*_*cm*_ < 3 will be noted as not supported. While 1/4 of total genes in the same pathway were associated with type E1 evidence, the query gene will be classified as strongly suggestive (*S*_*cm*_ ≥ 4.25). We obtained 677 genes that were strongly suggestive, 4391 genes that were suggestive, and 887 genes that were not supported with respect to being a HCC marker. Based on GO enrichment and KEGG analysis, the most significant gene functions of HCC markers were identified as follows: 4834 HCC markers (11.4%) were involved in Biological process, 4799 (15.7%) were involved in Cellular component; 4441 (45.1%) were involved in Binding of Molecular function, and 1157 (14.3%) were involved in Signal transduction pathways (Fig 2). These results are consistent with the known mechanism**s** of cancer development.

In dBMHCC, the average score from the HCC marker evaluation process was 3.745 (out of 10), and the top nine scores are shown in Table 2. The top three genes in Table 2 are the death-associated protein kinases *DAPK3*, *DAPK1*, and *DAPK2*. DAPK, a tumor suppressor, is a positive mediator of the p38 mitogen-activated protein kinase inhibitor SB203580 and thyroid hormone induction of autophagy of HCC cells [25, 26]. Tropomyosin-receptor kinase (TRK)-fused gene, *TFG*, is a novel HCC-related gene, which has a role in controlling cell size and regulating apoptosis and cell proliferation by fusing with different partners [27]. For example, TFG can fuse with neurotrophic tyrosine kinase receptor 1 (NTRK1), neuron-derived orphan receptor 1 (NOR1), and anaplastic lymphoma receptor tyrosine kinase (ALK) involving in extraskeletal myxoid chondrosarcoma, anaplastic large cell lymphoma, inflammatory myofibroblastic tumors, lung cancer, and neuroblastoma [28, 29]. These findings may indicate that TFG has high potential as an HCC marker. In addition, matrix metallopeptidase 1, *MMP1*, is another novel HCC-related gene. MMP1 is involved in the breakdown of extracellular matrix during normal and disease processes, and has been identified as a crucial factor for invasion and metastasis in bladder cancer, stomach cancer, breast cancer, and esophageal squamous cell carcinoma [30–33]. According to these results, the top three predicted HCC markers are involved in HCC, and the following six predicted markers are involved in other cancers. That is, dBMHCC did provide a reliable HCC marker prediction tool.

**Table 2:** Top nine candidates of HCC marker genes selected based on *S_cm_* score.

Gene ID	Gene Name	*S*_*cm*_
1613	*DAPK3*	5.37
1612	*DAPK1*	5.37
23604	*DAPK2*	5.37
10342	*TFG*	4.69
8030	*CCDC6*	4.69
8031	*NCOA4*	4.69
4312	*MMP1*	4.64
1870	*E2F2*	4.58
1871	*E2F3*	4.58

### HCC phosphorylated marker (HCCPM) evaluation

Phosphorylation and HCC relationship are two key factors of HCC phosphorylated marker (HCCPM) evaluation. Thus the scoring system was incorporated the evaluation scores of *S*_*k*_ and *S*_*cm*_, which were calculated by Eqs (1) and (4), respectively. Based on their classification criteria, if the query proteins were considered to be strongly suggestive in both correlation analysis of phosphorylation (*S*_*k*_ ≥ 6.8) and HCC marker evaluation (*S*_*cm*_ ≥ 4.25), it will be considered strongly suggestive in HCCPM evaluation. If the query proteins were considered to be not supported in both correlation analysis of phosphorylation (*S*_*k*_ < 4.8) and HCC marker evaluation (*S*_*cm*_ < 3), it will be considered to be not supported in HCCPM evaluation. While the scores were between the levels of strongly suggestive and not supported in HCCPM evaluation, the query protein will be considered to be suggestive. We obtained 19 genes that were strongly suggestive, 1261 genes that were suggestive, and 4675 genes that were not supported with respect to the evaluation scores *S*_*k*_ and *S*_*cm*_. Based on GO enrichment and KEGG analysis, the most significant gene functions of HCCPMs were identified as follows: 1241 HCCPMs (10.5%) were involved in Biological process, 1269 (15.8%) were involved in Cellular component, 1176 (47.3%) were involved in Binding of Molecular function, and 289 (14.4%) were involved in Signal transduction pathways (Fig 2). These results are consistent with the functional classification of HCC markers described above.

The top 10 HCCPMs are listed in Table 3. FK506 binding protein 51 (FKBP51, also called FKBP5), an immunophilin, has roles in signaling regulation, tumorigenesis, and chemoresistance [34]. FKBP5 is highly expressed in multiple tissues, including kidney, skeletal muscle, liver, placenta, heart, and peripheral blood. FKBP5 can stimulate the formation of a FKBP5-Hsp90-p23 superchaperone complex, which promotes cell growth in prostate cancer via androgen receptor-mediated signaling [35]. In contrast, downregulation of FKBP5 can promote apoptosis of lymphoblastic leukemia and irradiated melanoma cells through the activation of the transcription factor nuclear factor-κB (NF-κB) [34]. Considering that NF-κB regulates multiple essential functions in hepatocellular injury, liver fibrosis, and HCC [36], in addition to high expression of FKBP5 in the liver [37], we propose that FKBP5 may play a key role in HCC development through NF-κB signaling.

**Table 3:** Top 10 candidates for HCCPMs selected based on phosphorylation correlation analysis (*S*_*k*_) and pathway evaluation (*S*_*cm*_).

Gene ID	Accession Number^a^	Gene Name	*S*_*k*_	*S*_*cm*_
2289	Q13451	*FKBP5*	8.00	4.29
7175	P12270	*TPR*	7.40	4.26
8030	Q16204	*CCDC6*	6.00	4.69
83667	P58004	*SESN2*	6.00	4.53
55367	Q9HB75	*PIDD1*	6.00	4.53
64065	Q96FX8	*PERP*	6.00	4.53
55256	Q9BV57	*ADI1*	6.00	4.49
27430	Q9NZL9	*MAT2B*	6.00	4.49
2069	O14944	*EREG*	6.00	4.42
9252	O75582	*RPS6KA5*	6.00	4.36

^a^ UniprotKB/SwissProt Accession Number.

The second-highest candidate, TPR, a nucleoporin, was named based on its translocated promoter region to the kinase domain of the proto-oncogene MET. TPR translocation with MET is associated with an early step during carcinogenesis in gastric carcinoma [38]. TPR also undergoes translocation with NTrk1 (TrkA), a process that is associated with papillary thyroid carcinoma [39].

The third-highest candidate, CCDC6, participates in the function of the PTC1/ret proto-oncogene and is associated with papillary thyroid carcinomas and lung cancers [40, 41]. Phosphorylation of CCDC6, mediated by ataxia-telangiectasia mutated (ATM) kinase, prevents the FBXW7-CCDC6 interaction and FBXW7-mediated CCDC6 degradation during the DNA damage response [42]. Considering the high frequency of gene rearrangements of *CCDC6* in several cancers, and the *S*_*k*_ and *S*_*cm*_ evaluation in dBMHCC, *CCDC6* is a target gene worthy of further study with respect to its association with HCC.

Although acireductone dioxygenase 1 (*ADI1*) has not previously been shown to be a HCC-related gene, Chang et al. [43] found that overexpression of ADI1 regulates the infection of hepatitis C virus (HCV). HCV is well known as a leading etiology of HCC, and thus ADI1 might serve as a biomarker of HCV-mediated HCC.

Finally, methionine adenosyltransferase 2B (MAT2B) regulates HCC development. MAT2B interacts with GIT1 to increase ERK activity and growth in human liver and colon cancer cells [44]. According to these results, dBMHCC predicted several reliable HCCPMs. Based on these HCCPMs, dBMHCC can also provide their corresponding kinases, which could be used as the target for developing protein kinase inhibitors to improve survival and quality of life of cancer patients (7).

### Drug information

To provide HCCPM-related drug information, we collected 10,505 drugs and 16,804 targets from DrugBank 5.0.9 [2]. Among the HCCPMs, 9 genes were the targets of 5 liver cancer drugs, 91 genes were the targets of 124 cancer drugs, and 364 genes were the targets of 1515 non-cancer drugs. Platelet-derived growth factor receptor alpha, PDGFRA, which had the highest evaluation scores (*S*_*k*_ = 8, *S*_*cm*_ = 10) in dBMHCC, was the target of one HCC drug (Regorafenib), five cancer drugs (Imatinib, Sunitinib, XL820, Olaratumab, and Amuvatinib), and four non-cancer drugs (Becaplermin, Pazopanib, Midostaurin, and Ponatinib). Cyclin-dependent kinase 6, CD6, which was highly suggestive as a HCC marker in dBMHCC (*S*_*k*_ = 6, *S*_*cm*_ = 4.21), was the target of four cancer drugs (Alvocidib, Palbociclib, Ribociclib, and Abemaciclib), and two non-cancer drugs ((2S)-2-({6-[(3-amino-5-chlorophenyl)amino]-9-isopropyl-9H-purin-2-yl}amino)-3-methylbutan-1-ol, and 3,7,3',4'-tetrahydroxyflavone). Receptor-type tyrosine-protein kinase FLT3 can be used for the diagnosis and the determination of optimal therapy for myeloid leukemia [45]. In dBMHCC, FLT3 was predicted as an HCCPM (*S*_*k*_ = 6.2, *S*_*cm*_ = 3.697) and was the target of one liver cancer drug (Sorafenib), three cancer drugs (Sunitinib, Amuvatinib, and Tandutinib), and six non-cancer drugs (ABT-869, Midostaurin, Ponatinib, Brigatinib, Quizartinib, and Nintedanib). In addition, those cancer drugs that target HCCPMs, can be investigated with dBMHCC to determine their feasibility with respect to HCC treatment, which should effectively shorten the time needed for new HCC cancer drug development.

### Web interface

dBMHCC provides a user-friendly web interface that integrates HCC phosphor marker-related information and a prediction platform. Databases and Biomarker Prediction are the two main functional units in dBMHCC.

**Fig 3:**
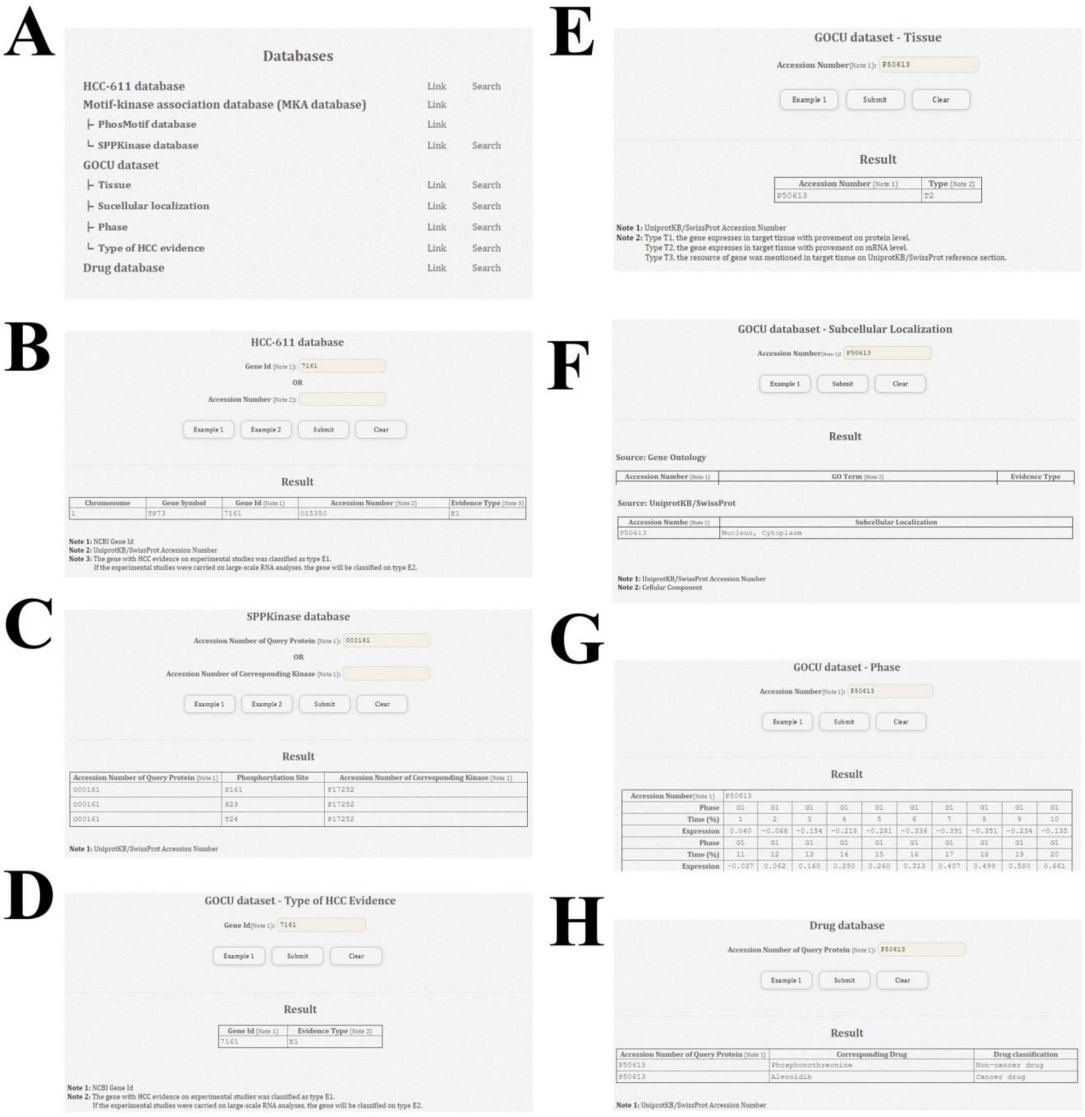
Web interface of search tools and results in the Databases unit of dBMHCC. (A) Web portal of the Databases unit in dBMHCC. dBMHCC contains the HCC-611, MKA, GOCU, and Drug databases and their sub-databases. Users can click Link to download the database information and click Search to investigate the related information for a query protein. (B–H) Web interfaces for the search tools and results from the HCC-611 database (B); the SPPKinase database (C); the type of HCC evidence (D), type of evidence for tissue-specific expression data (E), subcellular localization (F), and phase-specific expression during the cell cycle (G) from the GOCU database; and the Drug database (H).

In the Databases unit (Fig 3), the user can use the Link tool to retrieve information from the HCC-611, MKA, GOCU, and Drug databases (Fig 3A). The individual pages for the HCC-611 and SPPKinase databases are shown (Fig 3B and 3C), each of which includes a search function and provides the results from such searches. The other MKA database, PhosMotif, provides Prosite Documentation, Prosite Discriminator, Motif Name, Description, Motif Pattern, Max Repeat, Phosphorylation Site, and Skip Flag of Phosphorylation related Motif information based on a search query. Four GOCU-related results can be generated, HCC Evidence Type and the Tissue, Subcellular Localization, and Phase expression information (Fig 3D–3G). From the Drug database page, the user can use the Search tool to investigate the corresponding drug via the UniProtKB/SwissProt accession number of a protein of interest (Fig 3H).

**Fig 4:**
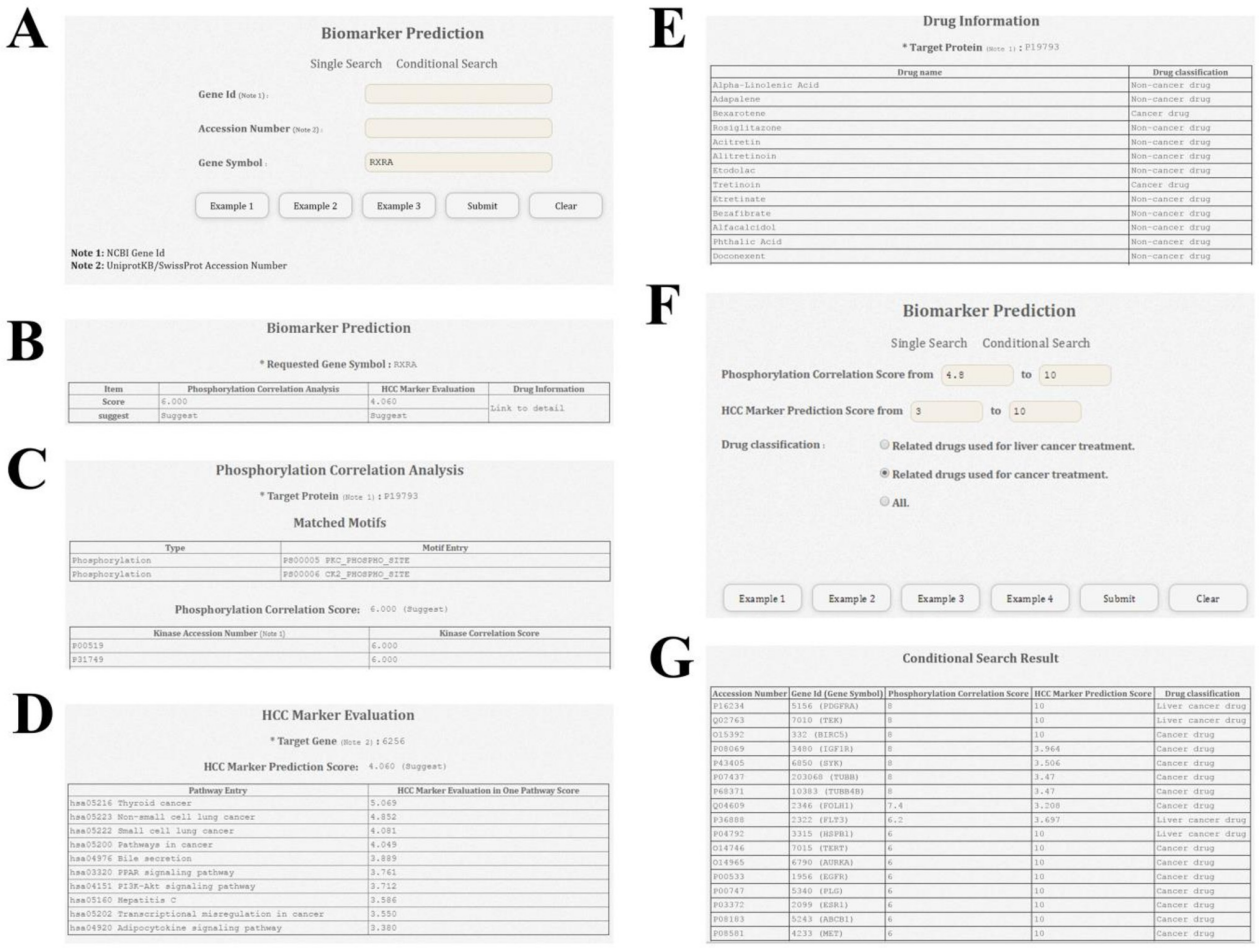
Web interface of the Biomarker Prediction unit of dBMHCC. (A) The dBMHCC website provides a Single Search and Conditional Search for HCCPMs. (B–E) The quick screen of overall information from the Biomarker Prediction (B) is shown first, with detailed information from the Phosphorylation Correlation Analysis (C), HCC Marker Evaluation (D), and Drug Information (E) displayed subsequently in sequence. The Conditional Search for Biomarker Prediction allows the use of up to three screening criteria (F). Based on a Conditional Search, dBMHCC will provide Accession Number, Gene ID, Gene Symbol, Phosphorylation Correlation Score, HCC Marker Prediction Score, and Drug classification of the qualified proteins (G). Clicking on the Accession Number, Gene ID, or Gene Symbol from the Conditional Search Result, will provide detailed information resulting from a Single Search.

In the Biomarker Prediction unit (Fig 4), both a Single and Conditional Search are available. The user can use the NCBI Gene ID, UniProtKB/SwissProt Accession Number, or the Gene Symbol for a Single Search (Fig 4A). The quick screen showing the overall information will help the user decide whether to carry out further searches or not (Fig 4B), and the detailed information for the Phosphorylation Correlation Analysis (Fig 4C), HCC Marker Evaluation (Fig 4D), and Drug Information (Fig 4E) pages will be displayed in sequence. For additional detailed information, the user can click on the Motif Entry value from the Phosphorylation Correlation Analysis page to obtain the Matched Pattern in the query protein and detailed information about that motif and its corresponding kinase. Clicking the Kinase Accession Number, the user will get the co-expression information about the target gene and corresponding kinase, such as the tissue, subcellular localization, and phase expression data, to investigate the possibility of phosphorylation. On the HCC Marker Prediction page, the user can click the Pathway Entry value to get detailed information about the HCC-related pathway, including the Pathway Name and all the participating genes. If the gene has been experimentally identified as an HCC-related gene, it will be shown in red. On the Drug Information page, clicking the Drug name, will take the user to the corresponding DrugBank entry for detailed information. To benefit systemic analyses, dBMHCC also provides a Conditional Search. The user can set the range for the Phosphorylation Correlation or HCC Marker Prediction Score or select the Drug classification to obtain the proteins that meet the screening criteria (Fig 4F). The resulting Accession Number, Gene Symbol, Phosphorylation Correlation Score, HCC Marker Prediction Score, and Drug classification data are then listed on the result page (Fig 4G). Clicking an Accession Number from this result page results in a Single Search.

## Conclusion

We have constructed dBMHCC, a comprehensive HCC biomarker database with a reliable prediction system for novel HCC phosphorylated biomarkers. dBMHCC contains 611 HCC significant genes, 234 HCC-related pathways, 17 phosphorylation-related motifs and their 255 corresponding protein kinases, 5955 HCC biomarkers, and 1077 predicted HCCPMs. Among the HCCPMs, 9 genes were the targets of 5 liver cancer drugs, 91 genes were the targets of 124 cancer drugs, and 364 genes were the targets of 1515 non-cancer drugs. The cancer drugs that target HCCPMs should be investigated for their feasibility in HCC treatment, which should effectively shorten the time needed to develop new HCC cancer drugs. In conclusion, dBMHCC is a free and open resource HCC biomarker database, which provides expression profiles, evidence type, and drug information, to support researchers investigating the development of HCC and designing novel HCC-related diagnostics and drug treatment.

## Supporting information

S1 Fig Gene expression information during phases of the cell cycle as provided by the GOCU database. It shows the GOCU-generated information for one entry (A0AVK6). The phase length, time scale, and expression level were given by Cyclebase (17). The phase length of cell cycle was determined by expression profiles of human periodic genes. The common time scale was chosen to be in percent of the cell division cycle with 100% corresponding to cytokinesis (M/G1-transition). The expression level was presented as log2 (Cy5-labled cDNA from synchronous cells/Cy3-labled cDNA from asynchronously growing HeLa cells), where (Cy5/Cy3) is the normalized ratio of the background-corrected intensities.
(PDF)

S1 Table Classification of subcellular localization evidence information obtained from Gene Ontology. (PDF)

S2 Table Information about HCC-related genes from the HCC-611 database. (PDF)

S3 Table Classification of expression data for tissue-specific expression of various genes. (PDF)

S4 Table Subcellular localization information obtained from Gene Ontology. (PDF)

S5 Table Subcellular localization information obtained from UniProtKB/SwissProt. (PDF)

S6 Table Information about the corresponding kinase and phosphorylation site for a query protein. (PDF)

S7 Table Number of corresponding kinases for each phosphorylation-related motif. (PDF)

S8 Table Top 13 genes involved in multiple pathways. (PDF)

S9 Table The number of pathways in which HCC-related genes are involved. (PDF)
