# Supplementary material for: dBMHCC: A comprehensive hepatocellular carcinoma (HCC) biomarker database provides a reliable prediction system for novel HCC phosphorylated biomarkers
Source: PLoS One. 2020 Jun 4;15(6):e0234084. doi: 10.1371/journal.pone.0234084 (PMC7272086; doi:10.1371/journal.pone.0234084)
Supplement: S1 Fig — It shows the GOCU-generated information for one entry (A0AVK6). The phase length, time scale, and expression level were given by Cyclebase (17). The phase length of cell cycle was determined by expression profiles of human periodic genes. The common time scale was chosen to be in percent of the cell division cycle with 100% corresponding to cytokinesis (M/G1-transition). The expression level was presented as log2 (Cy5-labled cDNA from synchronous cells/Cy3-labled cDNA from asynchronously growing HeLa cells), where (Cy5/Cy3) is the normalized ratio of the background-corrected intensities. (PDF) [file pone.0234084.s001.pdf]

GOCU dataset - Phase

Accession Number (Note 1)	A0AVK6									
Phase	G1	G1	G1	G1	G1	G1	G1	G1	G1	G1
Time (%)	1	2	3	4	5	6	7	8	9	10
Expression	0.651	0.652	0.621	0.559	0.500	0.467	0.434	0.391	0.338	0.305
Phase	G1	G1	G1	G1	G1	G1	G1	G1	G1	G1
Time (%)	11	12	13	14	15	16	17	18	19	20
Expression	0.273	0.241	0.208	0.185	0.243	0.302	0.362	0.421	0.478	0.536
Phase	G1	G1	G1	G1	G1	G1	G1	G1	G1	G1
Time (%)	21	22	23	24	25	26	27	28	29	30
Expression	0.533	0.499	0.508	0.517	0.526	0.534	0.553	0.614	0.645	0.650
Phase	G1	G1	G1	G1	G1	G1	G1	G1	G1	G1
Time (%)	31	32	33	34	35	36	37	38	39	40
Expression	0.643	0.607	0.571	0.418	0.226	0.062	-0.102	-0.266	-0.430	-0.530
Phase	G1	G1	G1	G1	G1	G1	G1/S	S	S	S
Time (%)	41	42	43	44	45	46	47	48	49	50
Expression	-0.476	-0.430	-0.391	-0.624	-0.657	-0.689	-0.934	-1.222	-1.564	-1.907
Phase	S	S	S	S	S	S	S	S	S	S
Time (%)	51	52	53	54	55	56	57	58	59	60
Expression	-2.249	-2.591	-2.889	-3.117	-3.289	-3.321	-3.334	-3.328	-3.322	-3.133
Phase	S	S	S	S	S	S	S	S	S	S/G2
Time (%)	61	62	63	64	65	66	67	68	69	70
Expression	-2.929	-2.694	-2.459	-2.223	-1.988	-1.880	-1.923	-1.947	-1.956	-1.951
Phase	G2	G2	G2	G2	G2	G2	G2	G2	G2	G2
Time (%)	71	72	73	74	75	76	77	78	79	80
Expression	-1.936	-1.917	-1.755	-1.593	-1.410	-1.227	-1.033	-0.832	-0.646	-0.471
Phase	G2	G2	G2	G2	G2	G2	G2	G2	G2	G2/M
Time (%)	81	82	83	84	85	86	87	88	89	90
Expression	-0.284	-0.087	0.101	0.286	0.470	0.655	0.838	1.033	1.237	1.441
Phase	M	M	M	M	M	M	M	M	M	M/G1
Time (%)	91	92	93	94	95	96	97	98	99	100
Expression	1.645	1.812	1.963	2.026	2.030	2.013	1.991	1.967	1.890	1.826

Note 1: UniprotKB/SwissProt Accession Number

Figure S1. Gene expression information during phases of the cell cycle as provided by the GOCU database. It shows the GOCU-generated information for one entry (A0AVK6)**.** The phase length, time scale, and expression level were given by Cyclebase (17). The phase length of cell cycle was determined by expression profiles of human periodic genes. The common time scale was chosen to be in percent of the cell division cycle with 100% corresponding to cytokinesis (M/G1-transition). The expression level was presented as $\log_2$ (Cy5-labled cDNA from synchronous cells/Cy3-labled cDNA from asynchronously growing HeLa cells), where (Cy5/Cy3) is the normalized ratio of the background-corrected intensities.
