# Supplementary material for: dBMHCC: A comprehensive hepatocellular carcinoma (HCC) biomarker database provides a reliable prediction system for novel HCC phosphorylated biomarkers
Source: PLoS One. 2020 Jun 4;15(6):e0234084. doi: 10.1371/journal.pone.0234084 (PMC7272086; doi:10.1371/journal.pone.0234084)
Supplement: S1 Table — (PDF) [file pone.0234084.s002.pdf]

Table S1 Classification of subcellular localization evidence information obtained from Gene Ontology

Experimental Evidence Code	Experimental Evidence Name	Evidence Type[a]
EXP	Inferred from Experiment	L1
IDA	Inferred from Direct Assay	L1
IPI	Inferred from Physical Interaction	L1
IMP	Inferred from Mutant Phenotype	L1
IGI	Inferred from Genetic Interaction	L1
IEP	Inferred from Expression Pattern	L1
ISS	Inferred from Sequence or Structural Similarity	L2
ISO	Inferred from Sequence Orthology	L2
ISA	Inferred from Sequence Alignment	L2
ISM	Inferred from Sequence Model	L2
IGC	Inferred from Genomic Context	L2
IBA	Inferred from Biological aspect of Ancestor	L2
IBD	Inferred from Biological aspect of Descendant	L2
IKR	Inferred from Key Residues	L2
IRD	Inferred from Rapid Divergence	L2
RCA	inferred from Reviewed Computational Analysis	L2
TAS	Traceable Author Statement	L3
NAS	Non-traceable Author Statement	L3
IC	Inferred by Curator	L4
ND	No biological Data available	L4
IEA	Inferred from Electronic Annotation	L4
NR	Not Recorded	L4

[a] Evidence Type: L1, experimental evidence; L2, computational analysis evidence; L3, curatorial statement evidence; and L4, automatically-assigned evidence.
