# Supplementary material for: dBMHCC: A comprehensive hepatocellular carcinoma (HCC) biomarker database provides a reliable prediction system for novel HCC phosphorylated biomarkers
Source: PLoS One. 2020 Jun 4;15(6):e0234084. doi: 10.1371/journal.pone.0234084 (PMC7272086; doi:10.1371/journal.pone.0234084)
Supplement: S2 Table — (PDF) [file pone.0234084.s003.pdf]

Table S2 Information about HCC-related genes from the HCC-611 database

Chromosome	Gene Symbol	Gene ID[a]	Accession Number[b]	Evidence Type[c]
1	*TP73*	7161	O15350	E1
1	*ENO1*	2023	P06733	E2
1	*MASP2*	10747	O00187	E2
1	*FNDC5*	252995	Q8NAU1-4[d]	E2
2	*ADAM17*	6868	P78536	E1
2	*RRM2*	6241	P31350	E2
2	*RHOB*	388	P62745	E2
3	*ROBO1*	6091	Q9Y6N7	E1
3	*TMEM45A*	55076	Q9NWC5	E2
3	*MUC13*	56667	Q9H3R2	E2
11	*H19*	283120	N/A[e]	E1
16	*WWOX*	51741	Q9NZC7-4[d]	E1
17	*TOP2A*	7153	P11388-2[d]	E1
17	*MIR22HG*	84981	N/A[e]	E2
X	*SSX1*	6756	Q16384	E1
X	*SSX4*	6759	O60224	E1
X	*RBM3*	5935	P98179	E2
Y	*TSPY1*	7258	Q01534	E1
Y	*RBMY1A1*	5940	P0DJD3	E1

[a] NCBI Gene ID

[b] UniProtKB/SwissProt accession number

[c] A gene with HCC evidence from small-scale experimental studies was classified as type E1. If the **evidence** consisted of large-scale RNA analyses, the gene was classified as type E2.

[d] Isoform

[e] Non-protein-coding gene
