# Supplementary material for: dBMHCC: A comprehensive hepatocellular carcinoma (HCC) biomarker database provides a reliable prediction system for novel HCC phosphorylated biomarkers
Source: PLoS One. 2020 Jun 4;15(6):e0234084. doi: 10.1371/journal.pone.0234084 (PMC7272086; doi:10.1371/journal.pone.0234084)
Supplement: S3 Table — (PDF) [file pone.0234084.s004.pdf]

Table S3. Classification of expression data for tissue-specific expression of various genes

Accession Number[a]	Type[b]
P09488	T1
Q15084	T1
Q9NWB7	T1
P53708	T1
Q8TDP1	T2
P58004	T2
Q13618	T2
O95881	T2
Q16760	T3
Q9BTZ2	T3
Q9NS23	T3
P21757	T3

[a] UniProtKB/SwissProt accession number

[b] Type T1, T2, or T3 indicates that the data for tissue-specific gene expression is based on protein assays, mRNA analyses, or the genes were obtained from the "Sequence origin" section of UniProtKB/SwissProt, without additional specification, respectively.
