# Supplementary material for: dBMHCC: A comprehensive hepatocellular carcinoma (HCC) biomarker database provides a reliable prediction system for novel HCC phosphorylated biomarkers
Source: PLoS One. 2020 Jun 4;15(6):e0234084. doi: 10.1371/journal.pone.0234084 (PMC7272086; doi:10.1371/journal.pone.0234084)
Supplement: S4 Table — (PDF) [file pone.0234084.s005.pdf]

Table S4. Subcellular localization information obtained from Gene Ontology

Accession Number[a]	GO Term[b]	Experimental Evidence Code[c]
A0A5B9	GO:0016021 integral to membrane	IEA
A0AV02	GO:0016021 integral to membrane	IEA
A0AV96	GO:0005634 nucleus	IEA
A0AVF1	GO:0072372 primary cilium	ISS
A0AVI2	GO:0016021 integral to membrane	IEA
A0AVI4	GO:0005575 cellular component	ND
A0AVI4	GO:0016021 integral to membrane	IEA
A0AVK6	GO:0005667 transcription factor complex	IEA
A0AVT1	GO:0005737 cytoplasm	IDA
A0FGR8	GO:0005886 plasma membrane	IEA
A0FGR8	GO:0016021 integral to membrane	IEA
A0FGR9	GO:0005886 plasma membrane	IEA
A0FGR9	GO:0016021 integral to membrane	IEA

[a] UniProtKB/SwissProt accession number.

[b] GO Term: only the sub-ontology, Cellular Component, of GO.

[c] Experimental Evidence Code: IEA, inferred from electronic annotation; ISS, inferred from sequence or structural similarity; IDA, inferred from direct assay; ND, no biological data available.
