# Supplementary material for: dBMHCC: A comprehensive hepatocellular carcinoma (HCC) biomarker database provides a reliable prediction system for novel HCC phosphorylated biomarkers
Source: PLoS One. 2020 Jun 4;15(6):e0234084. doi: 10.1371/journal.pone.0234084 (PMC7272086; doi:10.1371/journal.pone.0234084)
Supplement: S5 Table — (PDF) [file pone.0234084.s006.pdf]

Table S5. Subcellular localization information obtained from UniProtKB/SwissProt

Accession Number[a]	Subcellular Localization
P60323	Nucleus, Cytoplasm
P60321	Cytoplasm
Q9UGK8	Cytoplasm, Nucleus
Q9UGK3	Cytoplasm
Q9UGL1	Nucleus
Q8TCZ2	Cell membrane
Q9UGI8	Cytoplasm
Q9UGI0	Cytoplasm, Nucleus
Q9BRX8	Cytoplasm
Q8TCY9	Cytoplasm, Nucleus
Q8TCY5	Cell membrane, ER/Golgi

[a] UniProtKB/SwissProt accession number
