# Supplementary material for: dBMHCC: A comprehensive hepatocellular carcinoma (HCC) biomarker database provides a reliable prediction system for novel HCC phosphorylated biomarkers
Source: PLoS One. 2020 Jun 4;15(6):e0234084. doi: 10.1371/journal.pone.0234084 (PMC7272086; doi:10.1371/journal.pone.0234084)
Supplement: S6 Table — (PDF) [file pone.0234084.s007.pdf]

Table S6. Information about the corresponding kinase and phosphorylation site for a query protein

Accession Number[a] of Query Protein	Accession Number[a] of Corresponding Kinase(s)	Phosphorylation Site[b]
Q9UGK3	O60674, P12931, Q13882	Y250
Q8TDC3	Q15831	T189
P02686	Q8TAS1	S299
Q14493	P68400	T61
Q14493	P06493	T62
P00439	P17612	S16
O94776	P24941	S435
P35568	O14920, Q13535, P23443	S270
P35568	Q05655, O14920, P23443	S307
P35568	P45983, O14920	S312
P35568	P06213	Y612
P35568	P31749	S629
P35568	P06213	Y632
P35568	P28482, P23443	S636
P35568	P57059, Q9H0K1	S794
P35568	P06213	Y941
P35568	Q04759, Q05655, P05129, P23443, Q15418	S1101
P35579	P68400	S1943
Q8TDX7	Q8TD19	S195
Q9BR76	P17252, Q05513	S2

[a] UniProtKB/SwissProt accession number

[b] Phosphorylation site and its corresponding amino acid residue
