# Supplementary material for: dBMHCC: A comprehensive hepatocellular carcinoma (HCC) biomarker database provides a reliable prediction system for novel HCC phosphorylated biomarkers
Source: PLoS One. 2020 Jun 4;15(6):e0234084. doi: 10.1371/journal.pone.0234084 (PMC7272086; doi:10.1371/journal.pone.0234084)
Supplement: S7 Table — (PDF) [file pone.0234084.s008.pdf]

Table S7. Number of corresponding kinases for each phosphorylation-related motif

Motif Entry	Motif Entry Name	No. of Kinases
PS00004	CAMP_PHOSPHO_SITE	102
PS00005	PKC_PHOSPHO_SITE	146
PS00006	CK2_PHOSPHO_SITE	167
PS00007	TYR_PHOSPHO_SITE	65
PS00018	EF_HAND_1	5
PS00107	PROTEIN_KINASE_ATP	15
PS00211	ABC_TRANSPORTER_1	1
PS00223	ANNEXIN	1
PS00237	G_PROTEIN_RECEP_F1_1	1
PS00239	RECEPTOR_TYR_KIN_II	6
PS00415	SYNAPSIN_1	1
PS00469	NDP_KINASES	3
PS00479	ZF_DAG_PE_1	1
PS00827	MARCKS_2	5
PS00889	CNMP_BINDING_2	1
PS01159	WW_DOMAIN_1	2
PS01351	MAPK	1
