# Supplementary material for: dBMHCC: A comprehensive hepatocellular carcinoma (HCC) biomarker database provides a reliable prediction system for novel HCC phosphorylated biomarkers
Source: PLoS One. 2020 Jun 4;15(6):e0234084. doi: 10.1371/journal.pone.0234084 (PMC7272086; doi:10.1371/journal.pone.0234084)
Supplement: S8 Table — (PDF) [file pone.0234084.s009.pdf]

Table S8. Top 13 genes involved in multiple pathways

Gene ID	Gene Name	No. of Pathways
5594	*MAPK1*	69
5595	*MAPK3*	69
5290	*PIK3CA*	54
5604	*MAP2K1*	49
208	*AKT2*	49
3845	*KRAS*	45
5894	*RAF1*	42
5605	*MAP2K2*	36
7157	*TP53*	31
2778	*GNAS*	28
595	*CCND1*	27
107	*ADCY1*	27
1956	*EGFR*	25
