# Supplementary material for: dBMHCC: A comprehensive hepatocellular carcinoma (HCC) biomarker database provides a reliable prediction system for novel HCC phosphorylated biomarkers
Source: PLoS One. 2020 Jun 4;15(6):e0234084. doi: 10.1371/journal.pone.0234084 (PMC7272086; doi:10.1371/journal.pone.0234084)
Supplement: S9 Table — (PDF) [file pone.0234084.s010.pdf]

Table S9. The number of pathways in which HCC-related genes are involved

No. of Pathways	No. of Genes
1–10	337
11–20	35
21–30	10
31–40	2
41–50	4
51–60	1
61–69	2
